# Small Bowel Adenocarcinoma Masquerading as Crohn’s Disease

**DOI:** 10.7759/cureus.92797

**Published:** 2025-09-20

**Authors:** Se Yeon Kim, Brandon Wiggins, Daniel Ramirez, Mark Minaudo

**Affiliations:** 1 Department of Internal Medicine, Henry Ford Genesys Hospital, Grand Blanc, USA; 2 Department of Internal Medicine/Division of Gastroenterology, Henry Ford Genesys Hospital, Grand Blanc, USA

**Keywords:** crohn’s disease (cd), gastroenterology tumor, ileocecal region, small bowel adenocarcinoma (sba), small bowel tumors

## Abstract

Small bowel adenocarcinoma (SBA) is a rare gastrointestinal malignancy, with the majority of cases seen in the duodenum. SBA often poses a diagnostic challenge due to its non-specific presentation. A 46-year-old female smoker presented with a 6-week history of persistent abdominal pain and nausea. The initial workups, including computed tomography and colonoscopy, demonstrated evidence of suspected Crohn’s disease (CD). The histopathology of an ileocecal valve ulcer revealed invasive adenocarcinoma. Our case highlights the necessity of considering SBA in the differential diagnosis for patients exhibiting symptoms similar to Crohn's disease, as delays in diagnosis and treatment may result in disease progression and complications, including small bowel obstruction.

## Introduction

Small bowel cancer accounts for only 0.6% of all cancers in the U.S. and approximately 3% of all gastrointestinal malignancies [1-3]. Approximately 30-40% of small bowel cancers are classified as small bowel adenocarcinoma (SBA) [2]. SBA is predominantly localized in the duodenum (52-57%) and less frequently in the ileum (10-13%) [4], the exception being patients with Crohn’s disease (CD), where SBA is most commonly found in the ileum. SBA is difficult to diagnose due to its non-specific symptoms, low index of suspicion, and limited diagnostic tools, resulting in a poor prognosis [3]. The 5-year survival rate of SBA is approximately 35% [3,4]. SBA has the propensity to mimic CD in both clinical and radiological features. Double-balloon enteroscopy (DBE) demonstrates the highest diagnostic yield but is limited to specialized centers with the required expertise. While video capsule endoscopy (VCE) is useful in evaluating small bowel pathology, it is contraindicated when obstruction is suspected [5,6]. A case presentation illustrates this point, wherein a 46-year-old female with no history of CD presents with recurrent abdominal pain and altered bowel habits and was found to have an invasive SBA in the terminal ileum.

## Case presentation

We report a case of a 46-year-old Caucasian female with chronic obstructive pulmonary disease, tobacco use, obesity, and a family history of inflammatory bowel disease, who presented with a 6-week history of abdominal pain. At age 34, she underwent her first colonoscopy extending to the ileum due to anemia of unknown etiology, which identified mild internal hemorrhoids, five hyperplastic polyps, and one adenoma; all polyps were removed via polypectomy. She was advised to have a repeat colonoscopy in three years, but she was lost to follow-up.

Two weeks after the initial onset of abdominal pain, the patient was evaluated at an outside facility, where she was diagnosed with irritable bowel syndrome. She was prescribed dicyclomine, amitriptyline, and omeprazole. However, her symptoms persisted. Four weeks later, approximately six weeks after her initial symptom onset, she presented to our institution with persistent, intermittent, crampy, postprandial mid-abdominal pain accompanied by nausea and multiple episodes of non-bilious emesis. She also had daily bowel movements with variable consistency.

On physical examination, the patient was afebrile and well-developed. An abdominal examination showed mild tenderness to palpation in the right lower quadrant without rebound or guarding; the remainder of the exam was unremarkable. Laboratory evaluation was notable for leukocytosis with a white blood cell count of 15,000; erythrocyte sedimentation rate, C-reactive protein, fecal calprotectin, hemoglobin, and iron studies were within normal limits.

**Figure 1:**
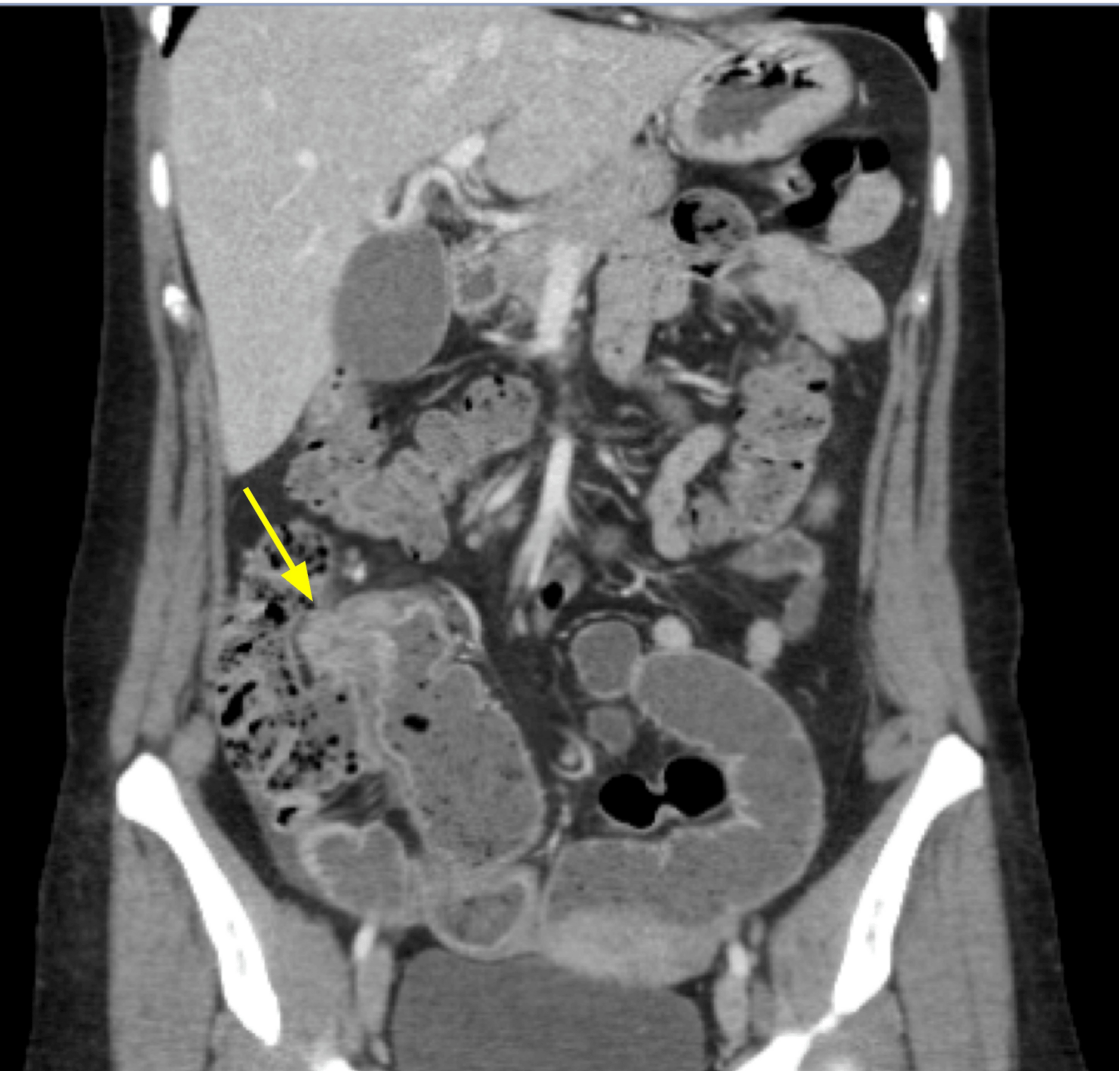
Contrast-enhanced CT scan of the abdomen and pelvis showed marked thickening, inflammation, and stricturing at the terminal ileum (yellow arrow) with multifocal mild to moderate strictures proximally. No fistulas, abscesses, or adenopathy were identified.

**Figure 2:**
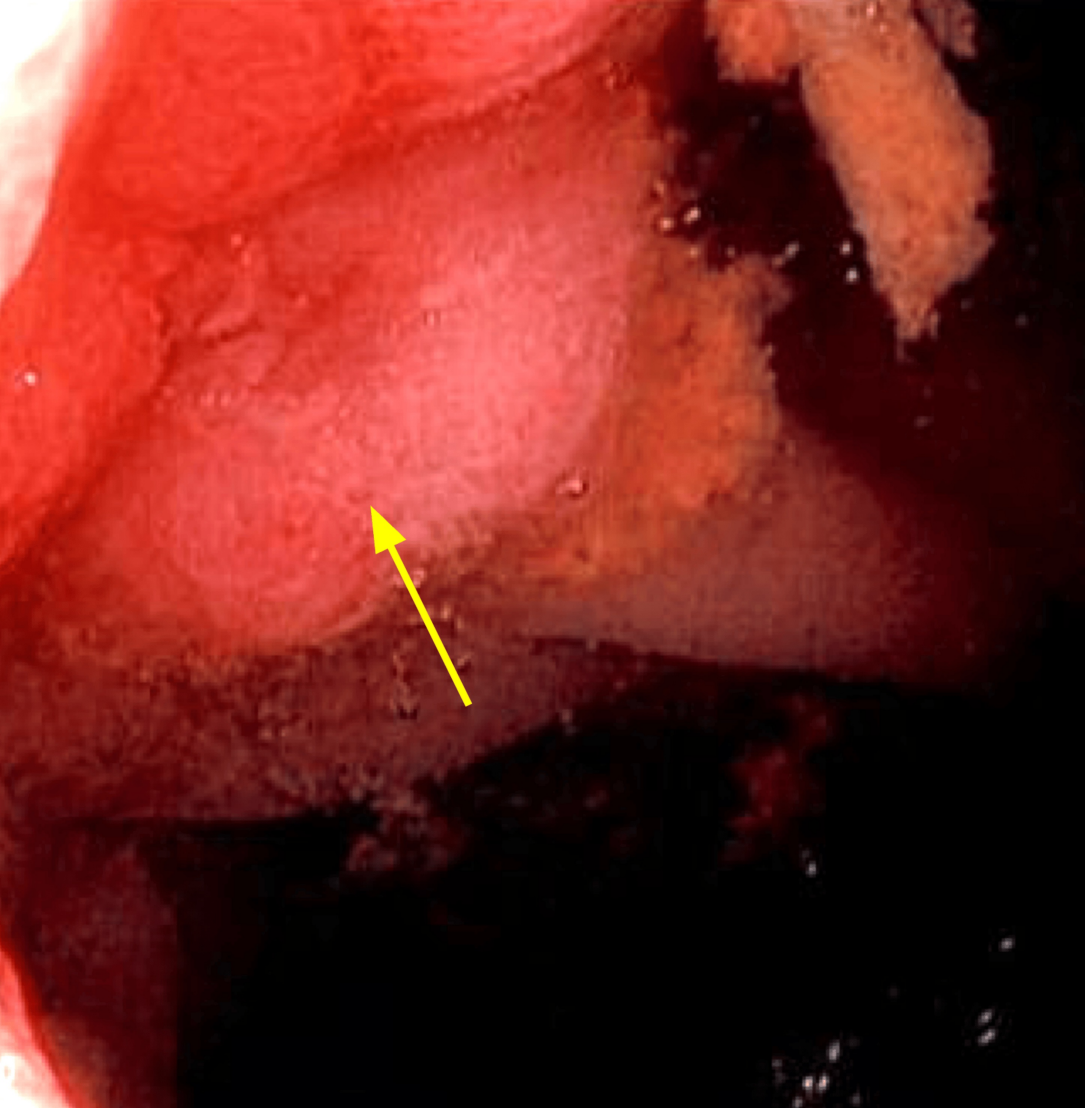
Colonoscopy revealed massive ulcerative lesions at the terminal ileum (yellow arrow), which prevented entrance into the ostium.

A contrast-enhanced CT scan of the abdomen and pelvis revealed marked thickening, strictures, and inflammation at the terminal ileum, with multifocal mild to moderate strictures proximal to this site. There was no evidence of fistula, abscess, or lymphadenopathy (Figure 1). The following day, an inpatient colonoscopy demonstrated ulcerative lesions measuring 3.6 x 3.0 cm at the terminal ileum, which precluded entrance into the ileocecal ostium (Figure 2). Although our patient had normal inflammatory markers, inflammatory bowel diseases, such as CD, could not be fully excluded from the differential diagnosis with the radiologic findings of multifocal areas with strictures and inflammation.

The patient’s management involved a multidisciplinary team, including gastroenterologists, general surgeons, oncologists, and pathologists. Within one week after discharge from our institution, she was readmitted with a small bowel obstruction and underwent right hemicolectomy and small bowel resection.

Pathological examination revealed a moderately differentiated invasive adenocarcinoma arising within a tubulovillous adenoma, centered at the ileocecal valve with extension into the terminal ileal mucosa, supporting a diagnosis of small bowel adenocarcinoma. Grossly, the 3.6 × 3.0 cm annular tumor displayed rolled borders and was transmural, invading into the pericecal adipose tissue and the visceral peritoneum. The resected specimen included 9.2 cm of terminal ileum and 9.4 cm of right colon, with no additional neoplasia identified in the colonic mucosa. Proximal and distal margins were negative. Histologic features included lymphovascular and perineural invasion and focally high tumor budding (≥10). Of 17 regional lymph nodes, 5 were positive for metastatic adenocarcinoma, including extracapsular extension. Immunohistochemical staining showed intact expression of all mismatch repair proteins, consistent with microsatellite stability. Final pathologic staging was pT4a N2a M0 (Stage III).

## Discussion

This paper describes an unusual presentation of SBA mimicking CD, leading to challenges in early diagnosis of SBA. Although the small bowel accounts for over 90% of the gastrointestinal mucosal surface area, the incidence of small bowel cancer is significantly uncommon, consisting of approximately 3% of all gastrointestinal malignancies [1,2,7]. This difference could be due to the unique nature of the small bowel, including its rapid movement, fluid content, alkaline environment, and high levels of immunoglobulin A. SBA comprises approximately 30 to 40% of SBC [2]. For most patients with SBA, the duodenum is the most frequent site of tumors, whereas the ileum is the least common location; however, for patients with CD, the ileum is the most common site for SBA [2,3]. 

Risk factors of SBA 

Modifiable risk factors of SBA closely resemble those of large bowel cancers, which include alcohol and tobacco use, a low-fiber diet, obesity, and high intake of processed foods [2]. Non-modifiable risk factors associated with SBA are African American race, age (median age of 66), history of inflammatory bowel disease, celiac disease, and hereditary mutations such as familial adenomatous polyposis and Lynch syndrome [2].

Diagnostic challenges

SBA poses a diagnostic challenge due to its vague symptoms, rarity of the disease, and limited diagnostic tools [7]. SBA is frequently diagnosed at an advanced stage, commonly presenting with symptoms of small bowel obstruction or gastrointestinal bleeding. As shown in our case, SBA can present similarly to CD. Our patient presents with vague abdominal pain, thickening of the bowel wall, segmental narrowing of the small intestine on imaging, and mucosal ulcers in the terminal ileum seen on colonoscopy. Both SBA and CD can result in vague gastrointestinal symptoms, weight loss, anemia, and similar radiologic findings such as strictures or bowel wall thickening. In contrast, SBA differs from CD as it typically involves a localized malignant mass that may lead to more progressive obstruction and predominantly occurs in older individuals without an extensive history of inflammatory bowel disease. These similarities may lead to a misdiagnosis of SBA, resulting in delayed correct treatment initiation. To improve early detection and, ultimately, the overall patient outcomes, it is crucial for healthcare providers to be aware of this condition and its propensity to mimic other GI pathologies.

Diagnostic modalities

Although a histopathology study is required for an accurate diagnosis of SBA, diagnostic modalities, such as videocapsule endoscopy (VCE) and double-balloon enteroscopy (DBE), may aid in the early detection of SBA. VCE can help visualize the entire small intestine in a non-invasive manner; yet, it should be avoided in cases where bowel obstruction is suspected, as it has the risk of capsule retention. Alternatively, DBE offers the highest sensitivity due to the direct visualization and its tissue sampling capability, but its availability is limited to specialized centers with experienced personnel [6].

## Conclusions

Over the last four decades, the incidence of SBC has increased by more than twofold. Several factors, such as the rise in obesity rates, the aging population, and advancements in diagnostic technologies, could contribute to this observed trend. Unfortunately, there is limited data on screening guidelines, diagnostic modalities, and optimal management strategies for SBA. Further research is needed to improve early detection and outcomes for patients with SBA. Our patient's small bowel adenocarcinoma had been mimicking Crohn’s disease for a prolonged period, resulting in a delay in surgical diagnosis that may have contributed to the development of small bowel obstruction. This case illustrates the value of maintaining a high index of suspicion for SBA in patients presenting with Crohn’s-like symptoms, particularly when the clinical course deviates from the typical presentation of inflammatory bowel disease.
